# A Comparative Study of Effectiveness of Splinting and Splinting Plus Local Corticosteroid Injection in Patients With Carpal Tunnel Syndrome: A Randomized Controlled Trial

**DOI:** 10.7759/cureus.52868

**Published:** 2024-01-24

**Authors:** Shikha LNU, Anurug Biswas

**Affiliations:** 1 Physical Medicine and Rehabilitation, Patna Medical College and Hospital, Patna, IND; 2 Physical Medicine and Rehabilitation, All India Institute of Medical Sciences, Patna, Patna, IND

**Keywords:** cts management, entrapment neuropathy, triamcinolone, cts splint, boston carpal tunnel questionnaire, corticosteroid injection, splint, carpal tunnel syndrome

## Abstract

Introduction

Corticosteroid injection and wrist-hand splint are two of the most commonly used conservative options for the management of carpal tunnel syndrome (CTS). This study compares the effectiveness of splinting and splinting plus local steroid injection in improving clinical and nerve conduction findings of patients with CTS.

Methods

A total of 44 patients with CTS were randomized into two groups. Group A used a full-time neutral wrist splint and group B was injected with 20 mg of triamcinolone acetonide and was given a full-time neutral wrist splint for 12 weeks. Clinical and nerve conduction findings of the patients were evaluated at baseline, 4 and 12 weeks after interventions. The chi-square test was used to test the association of different study variables. Z-test was used to test the significant difference between the two proportions. The means were compared by t-test. ANOVA was used to compare more than two mean values.

Results

The mean difference of the Boston Carpal Tunnel Questionnaire and median nerve latency at baseline and 12th week after treatment was significantly higher in group B than in group A (p<0.05). In intragroup comparison, there was significant improvement in the patient satisfaction, and clinical and nerve conduction values between the baseline level and 4 weeks after intervention and between the baseline and 12 weeks after intervention (P < 0.01). However, the inter-group comparisons were not significant.

Conclusion

Both of the management methods (splinting plus corticosteroid injection and splinting) have significant effects on the improvement of symptoms, and functional and nerve conduction status. It seems that splinting plus corticosteroid injection has a little edge over splinting alone during the follow-up periods.

## Introduction

Carpal tunnel syndrome (CTS) is one of the most common focal neuropathy and it is caused by compression of the median nerve at the wrist within the carpal tunnel. The prevalence of electro-physiologically confirmed symptomatic CTS is about 3% among women and 2% among men, with peak prevalence in middle-aged women [1]. CTS was first delineated in the medical literature in 1854 [2].

CTS hampers activities of daily living and reduces work capacity affecting quality of life and general health. CTS is characterized by pain, paresthesia, and numbness in the median nerve distribution (thumb, index finger, middle finger, and the radial side of the ring finger). It is usually worse at night causing sleep disruption, and may be alleviated by shaking the hand. It may result in a reduction in grip strength, and hand function [3,4].

CTS is usually a clinical diagnosis, made with the combination of a thorough history and physical examination in conjunction with the use of electro-diagnostic (EDX) testing for confirmation. Numbness in the distribution of the median nerve, nocturnal symptoms, thenar muscle weakness, atrophy, positive Tinel’s sign at the carpal tunnel, and abnormal sensory testing such as two-point discrimination have been standardized as clinical diagnostic criteria by consensus panels of experts [5,6].

Different conditions that can mimic CTS include cervical spondylosis, other median nerve pathology or median nerve compression at other locations, brachial plexopathy or neuritis, and other upper limb polyneuropathies. However, in rare instances, local compartment syndrome, fibromyalgia, degenerative conditions like osteoarthritis, inflammatory arthritis of the wrist and hand, etc. can produce similar symptoms like CTS [7].

Most cases of CTS are idiopathic as the exact reasons for the condition are unknown. Epidemiologic studies have identified several combinations of work factors, individual factors, and psychosocial factors related to CTS [4,8]. Female sex, obesity, pregnancy, and medical conditions including diabetes mellitus, thyroid disease, wrist osteoarthritis, and any form of inflammation affecting the wrist joints or tendon sheaths have been reported as a reason for increased risk of CTS [8,9]. Repetitive and forceful exertions of the hand, sustained awkward postures of the wrist, and use of vibrating hand tools are associated with an excess risk of CTS [8]. CTS is included in the list of so-called cumulative trauma disorders (CTD) [9].

Early diagnosis and treatment of CTS are important because delay can result in irreversible median nerve damage with persistent symptoms and permanent disability. Management approaches for CTS include various conservative and non-conservative methods. Though surgeries like carpal tunnel release have shown satisfactory symptomatic and functional outcomes, the acceptance of surgery in patients of CTS is poor, and nonsurgical methods are preferred over surgery [4,7,10]. A wide array of conservative options such as physical therapy, occupational therapy, orthosis, oral medications (non-steroidal anti-inflammatory drugs and corticosteroids), local injections of corticosteroids, platelet-rich plasma, electrotherapies like ultrasound therapy, and other non-conventional methods like acupuncture are used in the treatment of CTS [7,11,12]. Literature reviews regarding CTS indicate that soft to rigid splinting, and oral or local corticosteroids are a few of the most commonly used treatment methods but these methods usually do not offer long-term benefits [10].

However, there are no fixed guidelines or algorithms regarding conservative options due to heterogeneity of dose of injectable corticosteroids, and different splinting protocols used in different studies [12]. There is also a lack of long-term follow-up in previous studies [12].

It is an established norm that in the short term local perineural steroid injection has a slight edge over splinting but after a few weeks, the superiority between those two methods is debatable. However, splinting is more commonly advised than injectable procedures as splints are easily available and comparatively feasible with lesser side effects, especially when there is a lack of imaging guidance which is not uncommon in most parts of developing countries. The hypothesis behind our study is that a combination of both methods might show better outcomes than splinting alone, especially in longer duration.

The aim of this study was to compare the efficacy of splint and splint with local corticosteroid injection in improving clinical and nerve conduction of CTS patients.

## Materials and methods

Study type, location, setup, and duration

This prospective randomized controlled study was conducted in an apex institute of loco-motor disability situated in the eastern part of India from 2017 to 2019.

Ethical approval

The study complies with the ethical standard as per the declaration of Helsinki. Ethical approval was obtained from the concerned authority with clearance number, ‘IEC/1610/R&D/08/NIOH/68'.

Enrolment

After ethical approval, patients were enrolled as per inclusion criteria and randomized into two groups. A total of 44 patients satisfying the above criteria were enrolled in the study after giving properly signed informed consent.

Randomization

Randomization was done by the sealed envelope method and enrolled patients were equally distributed into either group A or group B.

Methodology

**Figure 1:**
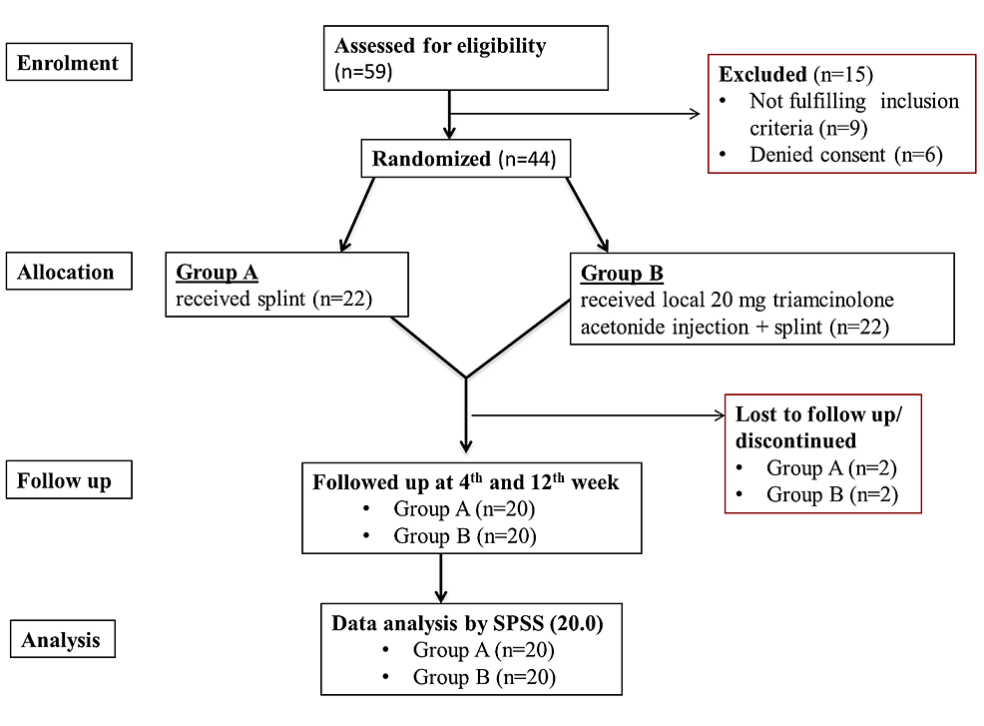
CONSORT diagram of the study CONSORT: Consolidated Standards of Reporting Trials

Patients in group A were advised to use a full-time static volar wrist splint for 12 weeks and patients in group B received surface marking guided perineural injection around the median nerve at the carpal tunnel with 20 mg triamcinolone acetonide (1 ml) and were prescribed to use full-time volar wrist static splint during the study period of 12 weeks. All the patients were advised ergonomic modifications and exercises which are commonly prescribed for CTS (active range of motion, wrist stretch, nerve/tendon gliding exercise, wrist flexion exercise, wrist extension exercise, and grip strengthening exercise). Patients were instructed not to take any NSAIDs. For occasional pain relief, oral opioid analgesics like tramadol (50 mg) were allowed when required. The Consolidated Standards of Reporting Trials (CONSORT) guideline was followed accordingly for reporting our study (Figure 1).

Fifty-nine patients were identified for the study. Of these, 50 patients were found to fulfill inclusion criteria, of which six patients denied consent. In group A, two patients discontinued intervention. In group B one patient discontinued intervention and one was lost to follow-up.

Inclusion criteria

Patients aged from 18 years to 60 years with clinical signs and symptoms of less than three months and electro-diagnostic evidence of CTS were included in the study.

Exclusion criteria

Patients with thenar muscle atrophy; with a history of cervical pathology, inflammatory arthritis, hypothyroidism, diabetes, coexisting systemic illness, malignancy, distal radius fracture; with a history of recent steroid injection, splinting, surgical management of CTS, pregnancy were excluded from the study.

Sample size

The sample size was calculated with the help of Epi Info (TM) 3.5.3. Epi Info is a trademarked software of the Centers for Disease Control and Prevention (CDC). With a power of 85% sample size was calculated to be 40 (20 in each group) based on the study by Mondelli et al. [13].

Method of injection

The procedure was performed in a minor operation theatre. Just medial to the palmaris longus tendon and proximal to the distal wrist crease, a 25 gauge needle was inserted slowly at a 45-degree angle with a direction along the middle finger and advanced about 1 to 2 cm. Following that 20 mg (1 ml) triamcinolone was injected. The needle was repositioned if there was any resistance to injection or any pain or paresthesia. Patients were instructed to limit extensive use of their wrists for the next 24 hours.

Splinting

A customized static volar wrist splint fabricated with low-temperature thermoplastic was applied to keep the wrist in neutral position to decrease the carpal tunnel pressure. The splint was prescribed for full-time (23 hours).

Outcome measures

Boston Carpal Tunnel Questionnaire (BCTQ)

BCTQ is a quantitative assessment of symptom severity and functional status that consists of symptom severity scale (SSS) and functional status scale (FSS) [14]. SSS consists of 11 questions with multiple-choice responses, scored from 1 point (mildest) to 5 points (most severe) and FSS consists of eight activities with similar scoring.

Electrophysiological Studies

Electrophysiological studies were performed with Neuropack® 2-MEB 7102-K device (Nihon Kohden, Japan) at a room temperature of 25 to 27 degree centigrade by the same technician for all the patients.

Median sensory and motor nerve conduction studies were performed on both hands of all the patients. Electrodiagnostic criteria for the diagnosis of CTS are as follows: median sensory velocity should be less than 50 m/sec, or median nerve distal motor latency (MNDML) should be higher than 4.2 m/sec [15]. Stainless steel surface disk electrodes were used for motor nerve conduction studies while ring electrodes were used for the antidromic sensory nerve conduction studies.

Statistical analysis

The data were tabulated in Microsoft® Excel, 2013, and later analyzed using SPSS Version 20.0 (IBM Corp., Armonk, NY, USA). The Chi-square test was used to test the association of different study variables. Z-test (Standard Normal Deviate) was used to test the significant difference between the two proportions. T-test was used to compare the means. One-way analysis of variance (ANOVA) was used to compare more than two mean values at a time and p < 0.05 was considered to be statistically significant.

## Results

This study was conducted on 40 patients divided equally into two groups. Baseline characteristics of the study population are mentioned in the following table (Table 1).

**Table 1:** Descriptive data of the two study groups SD: Standard deviation, BMI: Body Mass Index p<0.05: Significant

Different parameters	Group A (n=20)	Group B (n=20)	t-test	p-value
Age (in years)				
Mean±SD	38.60±8.33	40.45±6.94	0.76	0.45
Median	38.0	39.5		
Range	26 – 58	28 – 52		
BMI (in kg/m^2^)				
Mean±SD	24.75±2.49	24.67±2.47	0.82	0.41
Median	24.75	24.25		
Range	20.8 – 29.9	20.4 – 29.1		
Duration (in week)				
Mean±SD	9.35±2.60	8.45±3.13	0.98	0.33
Median	10	9.5		
Range	2 – 12	3 – 12		

T-test showed that there were no significant differences in mean age, BMI, and duration of disease of the patients of the two groups (p>0.05). A total of eight males and 32 females were included in the study. Group A consisted of two males and 18 females, and group B consisted of six males and 14 females. The corrected Chi-square test showed that there was no significant difference between the genders of the patients of the two groups (p=0.11). Patients of the two groups were also comparable in occupation. In group A, nine patients belonged to the lower-middle class and 11 patients belonged to the middle class. In group B, 12 patients belonged to the middle class, seven patients belonged to the lower-middle class and one patient belonged to the lower socio-economic strata. The corrected chi-square test showed that there was no significant difference between the socioeconomic status of the patients of the two groups (p=0.52). Only one out of 40 patients was left-handed (in group B). Fisher's exact test showed that there was no significant difference in the handedness of the patients of the two groups (p=0.05). Among 40 patients, 17 patients had involvement of the left hand (group A=9, group B=8) and 23 patients had involvement of the right hand (group A=11, group B=12). The chi-square test showed that there was no significant association between the side of the hand involved and patients of the two groups (p=0.74).

Corrected chi-square test showed that there was no significant association between patients’ satisfaction scores at presentation, 4th, and 12th weeks after interventions between the patients of the two groups (p>0.05) but intragroup comparison (at presentation, 4th and 12th weeks post-intervention) in both groups showed significant improvement in the satisfaction level at follow-up (Table 2).

**Table 2:** Satisfaction level of patients at different follow-up $\chi$^2^: chi square value *P<0.05: Significant

Likert scale		Time interval (in week)					
		At presentation (0 weeks)		At 4th week		At 12th week	
		Group-A	Group-B	Group-A	Group-B	Group-A	Group-B
Completely satisfied	Number	0	0	14	17	15	18
	Row %	0.0	0.0	45.2	54.8	45.5	54.5
	Col %	0.0	0.0	70.0	85.0	75.0	90.0
Almost satisfied	Number	0	0	5	3	4	2
	Row %	0.0	0.0	62.5	37.5	66.7	33.3
	Col %	0.0	0.0	25.0	15.0	20.0	10.0
Moderately satisfied	Number	0	0	1	0	1	0
	Row %	0.0	0.0	100.0	0.0	100.0	0.0
	Col %	0.0	0.0	5.0	0.0	5.0	0.0
Somewhat satisfied	Number	1	0	0	0	0	0
	Row %	100.0	0.0	0.0	0.0	0.0	0.0
	Col %	5.0	0.0	0.0	0.0	0.0	0.0
Dissatisfied	Number	19	20	0	0	0	0
	Row %	48.7	51.3	0.0	0.0	0.0	0.0
	Col %	95.0	100.0	0.0	0.0	0.0	0.0
Total	Number	20	20	20	20	20	20
	Row %	50.0	50.0	50.0	50.0	50.0	50.0
	Col %	100.0	100.0	100.0	100.0	100.0	100.0
$\chi$^2^		1.02		1.79		1.93	
p-value		0.31		0.40		0.37	

However, the test of proportion showed that the proportion of patients with complete satisfaction among the patients of Group B was significantly higher than that of the patients of Group A at the 4th week (Z=2.54; p=0.011).

For SSS, a comparison of the mean between time intervals (i.e., at presentation, at 4th week, and at 12th week) was made through one-way analysis of variance (ANOVA) followed by Tukey’s post hoc test (Table 3).

**Table 3:** Comparison of mean SSS and FSS of BCTQ of the patients of the two groups SSS: Symptom severity scale, FSS: Functional status scale, BCTQ: Boston Carpal Tunnel Questionnaire *p<0.05: Significant

BCTQ	Time interval (in week)					
	At presentation (0 weeks)		At 4th week		At 12th week	
SSS	Group-A	Group-B	Group-A	Group-B	Group-A	Group-B
Mean±SD	4.37±0.86	4.08±0.89	2.62±0.69	2.32±0.74	1.85±0.85	1.15±0.96
t-test	1.04		1.32		1.16	
p-value	0.30		0.19		0.28	
FSS	Group-A	Group-B	Group-A	Group-B	Group-A	Group-B
Mean±SD	4.41±0.72	4.57±1.12	1.65±0.72	1.55±0.74	1.57±0.73	1.54±0.74
t-test	1.81		0.43		0.12	
p-value	0.09		0.66		0.89	

There was a significant difference in the mean SSS of group A at the first presentation and at different follow-ups (F=11.73; p=0.0028) and also there was a significant difference in the mean SSS of group B at presentation at different follow-ups (F=10.10; p=0.0019). Thus the mean SSS improved (decreased) significantly at different follow-ups as compared to the first presentation for patients of both the groups. There were no significant differences in the mean SSS of the patients of the two groups at different time intervals (p>0.05). However, the mean SSS of the patients of group B was lower than that of the patients of group A at different follow-ups.

For FSS, a comparison of the mean between time intervals (i.e., at presentation, at 4th week, and at 12th week) was made through one-way analysis of variance (ANOVA) followed by Tukey’s post hoc test (Table 3). There was a significant difference in the mean FSS of group A at the first presentation and at different follow-ups (F=9.89; p<0.001) and there was a significant difference in the mean FSS of group B at presentation at different follow-ups (F=8.87; p<0.001). Thus the mean FSS improved (decreased) significantly at different follow-ups as compared to the first presentation for patients of both the groups. There were no significant differences in the mean FSS of the patients of both groups at different time intervals (p>0.05). However, the mean FSS of the patients of group B was lower than that of the patients of group A at different follow-ups.

The mean difference of SSS at baseline and at the 12th week after treatment was significantly higher in group B (2.93) than that of group A (2.52) (p<0.01). The mean difference of FSS at baseline and at the 12th week after treatment was significantly higher in group B (3.03) than that of group A (2.84).

According to the t-test, there were no significant differences in the mean of the combined sensory index (CSI) of the patients between the two groups at presentation and at the 12th week after treatment. However, the mean CSI at the 12th-week follow-up was significantly lower than that of at first presentation in both groups (intra-group comparison) (p<0.05) (Table 4).

**Table 4:** Comparison of CSI and MNDML of the patients of the two groups CSI: Combined sensory index, MNDML: Median nerve distal motor latency *p<0.05: Significant

	Variables	Baseline	After 12th week	t-test	p-value
CSI	Group-A	1.39±0.34	0.29±0.27	11.33	0.0001*
	Group-B	1.34±0.33	1.14±0.19	2.34	0.02*
	t-test	0.47	1.49		
	p-value	0.64	0.14		
MNDML	Group-A	6.22±1.38	5.45±1.46	1.71	0.09
	Group-B	5.85±1.42	4.78±1.17	2.60	<0.01*
	t-test	0.84	1.60		
	p-value	0.41	0.12		

Though the mean MNDML of the patients of both the groups decreased at the 12th week after treatment, the t-test showed that in intra-group comparison, there was no significant improvement for group A (p>0.05) but the improvement was significant for group B (p<0.01). According to the t-test, there were no significant differences in the mean MNDML of the patients between the two groups at presentation and at the 12th week after treatment. The mean pill count of group A was 2.8$\pm$1.642, and the mean pill count of group B was 1.95$\pm$1.504. The difference was non-significant.

## Discussion

In this comparative study between the splinting and splint with corticosteroid injection in CTS, a total of 40 patients completed the full 12 weeks of follow-up, 20 each in group A and group B. Patients of the two groups were matched for age, BMI, duration of disease, handedness, and laterality of the involved hand. They are also comparable in respect of occupation and socio-economic status. However, it is to be noted that three of our enrolled 44 individuals discontinued splinting after experiencing early improvement, and they did not follow the splinting protocol properly, and for that they were excluded from the study. One other individual did not visit on scheduled follow-up dates, and was excluded from the study.

Patient satisfaction improved in each follow-up in both groups (intragroup). However, patient satisfaction in each follow-up was not significantly different between the two study groups (intergroup) which is consistent with the previous study done by Khosrawi et al. [16].

The mean difference of SSS from baseline to the 12th week after treatment was significantly higher in Group B than in Group A. Also, the mean difference of FSS from baseline to the 12th week after treatment was significantly higher in Group B. Our study results were similar to the reported results of Khosrawi et al. [16].

Schmid et al. [17] found that the use of a splint can effectively reduce intra-neural edema and carpal tunnel pressure in mild to moderate carpal tunnel syndrome. Locally injected steroid also reduces edema and controls inflammation effectively in CTS [18]. It can reduce the generation of local inflammatory response, and inhibit extravasations of fluid, and it also has membrane stabilizing and analgesic properties by reduction of neural firing [19,20]. Simultaneous application of both of the management methods can have an additive effect in CTS. However, in this study, no statistically significant difference can be found between the two groups in inter-group comparison at different time points.

This study showed no significant difference in CSI change (from presentation to the 12th week after treatment) between group A and group B (p>0.05). However, the mean differences of MNDML at presentation and at the 12th week after treatment of group B were significantly higher than that of group A (p<0.05). Our findings were similar to the results of Khosrawi et al. [16]. Gupta et al. [21] found significant improvement in MNDML at one-month follow-up after steroid injection in CTS.

In this study, volar wrist static splint in a neutral position was used which is a common splinting method. There are different wrist-hand splints available for CTS management such as soft splint, volar cock up wrist splint, and modified ulnar gutter splint. However, as per recent evidence, most of the other splints do not match the benefits of the neutral splint [22]. Werner et al. [23] conducted a randomized controlled trial of nocturnal splinting for active workers with symptoms of carpal tunnel syndrome and found that a short course of nocturnal splinting may reduce wrist, hand, and/or finger discomfort among active workers with symptoms consistent with CTS. However, in this study, the duration of splinting was around 23 hours per day. Walker et al. [24] studied night-time versus full-time splinting in CTS and found that full-time neutral splinting is more beneficial than only night-splinting. Also, the rigid splint provides more immobilization than soft splints, thus it provides a better outcome than soft splints. In our study, splints were used for 12 weeks. Gatheridge et al. [25] found that beyond six weeks of splint usage, the improvement parameters hit a plateau. However, there is not enough evidence available presently regarding the optimal duration of splinting in CTS [25].

Agarwal et al. [26] conducted a prospective study of the long-term efficacy of local methylprednisolone acetate injection in the management of mild carpal tunnel syndrome and found that local glucocorticoid injection results in long-term improvement in nerve conduction parameters, symptom severity and functional scores in patients with mild CTS and the improvement persists even after 12 months. However, the present study assessed and followed patients up to 12 weeks. Methylprednisolone, triamcinolone, and β-methasone are commonly used corticosteroids in CTS. However, the improvement does not depend upon the dose of steroid as per available literature [27]. Also, the different needle entry points like at the wrist crease and distal to the wrist crease have no effect on symptomatic and electrophysiological improvement [27]. Present evidence favors the ultrasound-guided approach over landmark landmark-guided one. However, the safety profiles of these two approaches are comparable [28]. Ustün et al. [28] found better outcome in the ultrasound-guided injection group and there was a greater difference in improvement in the early phase than in later follow-ups suggesting superior outcome due to better localization through ultrasound. However, in the present study, surface marking guided injection technique was used. Most of the setups in developing countries do not have a facility for ultrasound-guided procedures compelling the physicians to adopt surface marking-guided approach. Sevim et al. [29] studied the effect of local injection of corticosteroid and orthosis for up to one year and they found that both methods have beneficial effects on symptomatic relief and electrophysiological findings. Furthermore, their study result also suggested that local steroid gives better outcome in the short term. At long-term follow-up, splinting can take the upper hand in improvement in CTS [29]. Though there are multiple studies regarding splinting versus local steroid injection in CTS, studies describing the effect of their combined use are still scarce.

In the present study, both methods offered a beneficial effect on the clinical and electrophysiological findings in the CTS patients. Considering some of the findings of the present study regarding SSS, FSS, and MNDML, it is suggested that splinting plus corticosteroid have a little better impact than splinting only. However, for obtaining more conclusive results, studies with large sample size is recommended. Moreover, a study regarding different dosage and dosing schedule of local corticosteroid with a long follow-up would be more helpful in this regard.

Limitation of study

As the treatment received by the patients was well obvious, blinding of the subjects could not be done. However, assessment was done by blinded interviewer reducing assessor-related bias. As our study period is 12 weeks only, the steroid plus splinting group might have shown a slightly better outcome than the splint-only group. As the effect of steroid weans off gradually, long-term follow-up might have suggested differently. The sample size of this study was small. This study also did not assess any correlation of comorbidities and other factors with outcome. Also, groups were not matched according to severity and that might have had an impact on outcome.

## Conclusions

In this study, the effect of neutral splint versus splinting along with local steroid injection on symptoms and functional status of patients with CTS was investigated. The electrophysiological improvement in both study groups was also analyzed. The results indicated that both methods have significant effects on patient satisfaction, symptoms, functional status, and nerve conduction findings of the studied patients. It is evident that combination therapy has a slight edge on SSS, FSS, and MNDML, especially during the follow-up periods. Both methods carry a good safety profile. Studies comparing two different stand-alone methods for the management of carpal tunnel syndrome are plenty in the literature but studies regarding combination of methods are still rare. That is why a study with a combination of different management methods is recommended in the future to achieve the highest level of benefit in CTS.

## References

[1] Atroshi I, Gummesson C, Johnsson R, Ornstein E, Ranstam J, Rosén I (1999). Prevalence of carpal tunnel syndrome in a general population. JAMA.

[2] Paget J (1854). Lectures on Surgical Pathology: Delivered at the Royal College of Surgeons of England. Philadelphia: Lindsay, Blakiston 1854.

[3] Spies-Dorgelo MN, van der Windt DA, van der Horst HE, Prins AP, Stalman WA (2007). Hand and wrist problems in general practice--patient characteristics and factors related to symptom severity. Rheumatology (Oxford).

[4] Aroori S, Spence RA (2008). Carpal tunnel syndrome. Ulster Med J.

[5] Graham B, Regehr G, Naglie G, Wright JG (2006). Development and validation of diagnostic criteria for carpal tunnel syndrome. J Hand Surg Am.

[6] Giacomini PS (2006). Electromyography and neuromuscular disorders: clinical electrophysiologic correlations. Mcgill J Med.

[7] Kleopa KA (2015). Carpal tunnel syndrome. Ann Intern Med.

[8] Stevens JC, Beard CM, O'Fallon WM, Kurland LT (1992). Conditions associated with carpal tunnel syndrome. Mayo Clin Proc.

[9] Lampainen K, Shiri R, Auvinen J, Karppinen J, Ryhänen J, Hulkkonen S (2022). Weight-related and personal risk factors of carpal tunnel syndrome in the Northern Finland Birth Cohort 1966. J Clin Med.

[10] Carlson H, Colbert A, Frydl J, Arnall E, Elliot M, Carlson N (2010). Current options for nonsurgical management of carpal tunnel syndrome. Int J Clin Rheumtol.

[11] Piazzini DB, Aprile I, Ferrara PE (2007). A systematic review of conservative treatment of carpal tunnel syndrome. Clin Rehabil.

[12] Khosrawi S, Moghtaderi A, Haghighat S (2012). Acupuncture in treatment of carpal tunnel syndrome: a randomized controlled trial study. J Res Med Sci.

[13] Mondelli M, Giannini F, Giacchi M (2002). Carpal tunnel syndrome incidence in a general population. Neurology.

[14] Levine DW, Simmons BP, Koris MJ, Daltroy LH, Hohl GG, Fossel AH, Katz JN (1993). A self-administered questionnaire for the assessment of severity of symptoms and functional status in carpal tunnel syndrome. J Bone Joint Surg Am.

[15] Rosario NB, De Jesus O (2023). Electrodiagnostic Evaluation of Carpal Tunnel Syndrome. https://www.ncbi.nlm.nih.gov/books/NBK562235/.

[16] Khosrawi S, Emadi M, Mahmoodian AE (2016). Effectiveness of splinting and splinting plus local steroid injection in severe carpal tunnel syndrome: a randomized control clinical trial. Adv Biomed Res.

[17] Schmid AB, Elliott JM, Strudwick MW, Little M, Coppieters MW (2012). Effect of splinting and exercise on intraneural edema of the median nerve in carpal tunnel syndrome--an MRI study to reveal therapeutic mechanisms. J Orthop Res.

[18] InformedHealth.org [Internet] (2014). Carpal Tunnel Syndrome: How Effective Are Corticosteroid Treatments?. https://www.ncbi.nlm.nih.gov/books/NBK279598/.

[19] Dworkin RH, O'Connor AB, Backonja M (2007). Pharmacologic management of neuropathic pain: evidence-based recommendations. Pain.

[20] Li H, Xie W, Strong JA, Zhang JM (2007). Systemic antiinflammatory corticosteroid reduces mechanical pain behavior, sympathetic sprouting, and elevation of proinflammatory cytokines in a rat model of neuropathic pain. Anesthesiology.

[21] Gupta S, Tewari AK, Nair V, Gupta A (2013). Reliability of motor parameters for follow-up after local steroid injection in carpal tunnel syndrome. J Neurosci Rural Pract.

[22] De Angelis MV, Pierfelice F, Di Giovanni P, Staniscia T, Uncini A (2009). Efficacy of a soft hand brace and a wrist splint for carpal tunnel syndrome: a randomized controlled study. Acta Neurol Scand.

[23] Werner RA, Franzblau A, Gell N (2005). Randomized controlled trial of nocturnal splinting for active workers with symptoms of carpal tunnel syndrome. Arch Phys Med Rehabil.

[24] Walker WC, Metzler M, Cifu DX, Swartz Z (2000). Neutral wrist splinting in carpal tunnel syndrome: a comparison of night-only versus full-time wear instructions. Arch Phys Med Rehabil.

[25] Gatheridge MA, Sholty EA, Inman A, Pattillo M, Mindrup F, Sanderson DL (2020). Splinting in carpal tunnel syndrome: the optimal duration. Mil Med.

[26] Agarwal V, Singh R, Sachdev A, Wiclaff Wiclaff, Shekhar S, Goel D (2005). A prospective study of the long-term efficacy of local methyl prednisolone acetate injection in the management of mild carpal tunnel syndrome. Rheumatology (Oxford).

[27] Boyer MI (2008). Corticosteroid injection for carpal tunnel syndrome. J Hand Surg Am.

[28] Ustün N, Tok F, Yagz AE (2013). Ultrasound-guided vs. blind steroid injections in carpal tunnel syndrome: a single-blind randomized prospective study. Am J Phys Med Rehabil.

[29] Sevim S, Dogu O, Camdeviren H, Kaleagasi H, Aral M, Arslan E, Milcan A (2004). Long-term effectiveness of steroid injections and splinting in mild and moderate carpal tunnel syndrome. Neurol Sci.

